# Application of Metal Oxide Nanoparticles for the Bacterial Transformation of (−)-Isopulegol

**DOI:** 10.3390/molecules31193445

**Published:** 2026-09-27

**Authors:** Polina Y. Maltseva, Alexandra A. Chudinova, Natalia A. Plotnitskaya, Irina B. Ivshina

**Affiliations:** Institute of Ecology and Genetics of Microorganisms of the Ural Branch of the Russian Academy of Sciences, Perm Federal Research Center of the Ural Branch of the Russian Academy of Sciences, 13 Golev Str., 614081 Perm, Russia; inbox.98@bk.ru (P.Y.M.); 79194890159@yandex.ru (A.A.C.); ivshina@iegm.ru (I.B.I.)

**Keywords:** biotransformation, (−)-isopulegol, metal oxide nanoparticles, *Rhodococcus rhodochrous*, terpenoid

## Abstract

**Background**: The biotransformation of the monoterpenoid (−)-isopulegol by *Rhodococcus rhodochrous* yields valuable diol and hydroxy acid derivatives; however, the yields are moderate, and formation of the (*S*)-epoxide byproduct significantly reduces the overall selectivity. Metal oxide nanoparticles (NPs) can modulate biocatalytic activity; however, their effects on monoterpenoid biotransformation are poorly understood. **Methods**: We investigated the effects of NiO (50 mg/L) and CuO NPs (5 mg/L) on (−)-isopulegol conversion by *R. rhodochrous* IEGM 1362. Products were quantified by GC–MS; cellular responses were assessed using respirometry, zeta potential measurements, AFM-CLSM, TEM, SEM, EDX, and cell viability assays. **Results**: NiO NPs at 50 mg/L significantly increased the total yield of target compounds (diol and hydroxy acid) from 36.1% to 48.3%. Importantly, NiO suppressed the formation of the undesired (*S*)-epoxide. In contrast, CuO NPs (5 mg/L) completely abolished target product formation. Both NPs reduced cell respiratory activity, shifted zeta potential toward less negative values, and induced cell aggregation and ultrastructural damage. NiO NPs were adsorbed onto the cell surface. **Conclusions**: NiO NPs selectively enhance the yield of valuable oxygenated metabolites of (−)-isopulegol while inhibiting the competing epoxidation pathway. Conversely, CuO NPs are detrimental and unsuitable for this bioprocess.

## 1. Introduction

The monoterpene alcohol (−)-isopulegol is among the terpenoids that are widely employed in the synthesis of bioactive substances. Its low cost, availability, and diverse biological properties have attracted considerable research interest in this compound [1]. The chemical transformation of (−)-isopulegol has already yielded derivatives with pronounced antiviral [2,3], analgesic [4], and antiproliferative [1] activities.

Currently, available information is limited to fragmentary reports on the transformation of (−)-isopulegol by *Aspergillus oryzae* and *Humicola insolens* [5], as well as on its whole-cell degradation by *Rhodococcus* sp. [6]. However, the literature lacks data on the specific metabolites arising from microbial transformation and their biotechnological potential. In this context, actinomycetes of the genus *Rhodococcus* are of particular interest because they exhibit pronounced catalytic activity in the transformation of complex hydrophobic compounds [7]. The strain *R. rhodochrous* IEGM 1362 was previously selected for its ability to convert (−)-isopulegol into the oxidation products Me-7-(1*R*,2*S*,5*R*)-5-(hydroxymethyl)-2-(prop-1-en-2-yl)cyclohexanol (diol **2**) and (1*R*,3*R*,4*S*)-3-hydroxy-4-(prop-1-en-2-yl)cyclohexanecarboxylic acid (hydroxy acid **3**) (Figure 1) [8]. Antitumor and analeptic activities have been predicted for both compounds. However, the total yield of the target products was only 25% (9.34% for diol **2** and 15.38% for hydroxy acid **3**). We attempted to develop biocatalysts based on poly(vinyl alcohol) cryogel with entrapped cells of *R. rhodochrous* IEGM 1362 in order to optimize and intensify the biotransformation of (−)-isopulegol [9]. The use of these biocatalysts increased the yield of the target compounds and enabled a continuous process for up to 13 substrate addition cycles. However, this bioconversion was accompanied by the formation of 30% or more of a (−)-isopulegol oxidation by-product, (*S*)-epoxide (**4**, Figure 1), which significantly reduced the process selectivity.

A promising approach to enhancing the selectivity of biocatalytic processes and increasing the yield of target products is the use of nanoparticles (NPs) of variable-valence metals (cobalt, nickel, copper, zinc, etc.) and their oxides. These materials are produced on a multiton scale and are widely used in industry and agriculture, leading to their release into the environment [10,11,12,13]. Owing to their physicochemical properties, NPs can exert complex effects on microbial cells: they can reversibly alter cell envelope permeability, serve as a source of cofactor ions for redox enzymes, and trigger adaptive stress responses [14,15,16,17,18,19,20]. This multifactorial action makes NPs an attractive tool for optimizing the biotransformation of hydrophobic monoterpenoids, whose conversion rates are often limited by substrate mass transfer. Nevertheless, information on the targeted application of NPs in the microbial transformation of terpenoids remains extremely scarce. The only detailed example is the conversion of the sesquiterpene alkaloid dendrobine using chemically synthesized gold nanoparticles (CH-AuNPs) and the fungus *Trichoderma longibrachiatum* MD33, in which the yield of the target compound increased by 67.3% [21]. The same NPs were previously successfully employed to enhance the synthesis of secondary terpene metabolites in plant cell cultures [22,23].

The choice of NiO and CuO NPs as modulators of the monoterpenoid biotransformation process in this work was based on their complementary physicochemical and biochemical mechanisms. A key factor limiting the conversion rate of hydrophobic substrates such as (−)-isopulegol is their restricted mass transfer across the cell envelope. According to Khashan et al. (2016), NiO NPs can reversibly increase the bacterial cell wall permeability, potentially intensifying the diffusion of (−)-isopulegol to intracellular enzyme systems [17]. In addition, CuO NPs can play a stabilizing role by inducing moderate oxidative stress in cells, which, as shown by Kuyukina et al. (2025), activates the extracellular polymeric matrix biosynthesis and promotes the formation of structurally organized biofilms [14]. The immobilization of NPs in such a matrix can shield the cells from direct cytotoxic effects while also simultaneously improving the operational stability of the biocatalyst.

The aim of this study was to investigate the effects of NiO and CuO NPs on *R. rhodochrous* IEGM 1362 cells and to evaluate their potential to enhance the bacterial transformation of (−)-isopulegol.

## 2. Results and Discussion

To determine the maximum concentrations of metal oxide NPs that can be used in biotransformation experiments, we first determined their MIC values under standard short-term screening conditions, which were 780 mg/L for NiO NPs and 195 mg/L for CuO NPs. However, these values were obtained in a static 24 h assay with low inoculum density and do not reflect the prolonged shaken cultivation with high cell density and substrate addition used in the biotransformation process. Therefore, we empirically selected sub-inhibitory concentrations of 50 mg/L NiO NPs and 5 mg/L CuO NPs based on preliminary viability assays under process-relevant conditions, where these concentrations allowed stable cell functionality throughout the cultivation period. Thus, these concentrations were used in further experiments.

Cultivation of bacterial cells in the presence of (−)-isopulegol and NiO and CuO NPs resulted in a decrease in the conversion of the starting substrate (Figure 2).

The addition of NiO NPs to the medium reduced the overall substrate conversion, but it increased the total yield of the target compounds (diol and hydroxy acid) from 36.1% to 48.3%, and, importantly, decreased the yield of the undesirable by-product—(*S*)-isopulegol epoxide (Figure 2). The observed increase in the yield of target products **2** and **3** in the presence of NiO NPs, accompanied by suppression of epoxide **4** formation, may indicate a redirection of metabolic fluxes. We propose that adsorption of NiO NPs onto the cell surface (the corresponding results are presented below) alters the local microenvironment and may facilitate the delivery of the hydrophobic substrate to membrane-associated oxygenases. Second, the moderate oxidative stress induced by NiO could trigger adaptive responses in *Rhodococcus* cells, which are likely to reflect upregulating detoxifying and hydroxylating enzyme systems and a partial suppression of epoxide-forming pathways. Third, the observed effect could potentially be attributed to the release of Ni^2+^ ions, which may exert cofactor effects on oxidoreductases, as previously reported for other microbial systems [20,24]. Although the precise molecular targets remain to be identified, our data collectively indicate that NiO NPs selectively modulate (−)-isopulegol biotransformation towards the desired hydroxylated products.

In contrast to NiO, CuO NPs completely abolished the formation of target products **2** and **3**, and only (*S*)-epoxide isopulegol was detected (Figure 2). Interestingly, (−)-isopulegol itself, like other monoterpenoids, can potentially act as a reducing and stabilizing agent in the synthesis of Cu NPs [25], which may, in the long term, lead to the formation of organomineral hybrid structures with enhanced colloidal stability. However, under our experimental conditions, the cytotoxic effect of CuO NPs appears to have offset these potential advantages.

Given the toxic effect of metal oxide NPs on bacterial cells, it is pertinent to evaluate their impact on *R. rhodochrous* IEGM 1362 during the biotransformation of (−)-isopulegol. Investigation of the responses of *Rhodococcus* to the presence of metal oxide NPs revealed that they do not exert a significant effect on the morphometric parameters of bacterial cells (Table 1). It was previously shown that (−)-isopulegol at a concentration of 0.025% has no cytotoxic effect and does not cause significant changes in cell morphometry [8]. In the present study, the substrate-only condition confirmed these data: the cell area was 6.19 ± 1.92 µm^2^ with an S/V ratio of 2.01 ± 0.37, which is comparable to the biotic control (5.56 ± 2.59 µm^2^ and 3.01 ± 1.11, respectively). Therefore, all morphometric changes observed upon the combined addition of NPs and substrate should be attributed specifically to the action of NiO or CuO NPs.

The addition of NiO NPs alone led to a reduction in cell size (area 3.32 ± 1.24 µm^2^, S/V = 2.88 ± 0.45), which is characteristic of a bacterial stress response. Upon co-cultivation with (−)-isopulegol, this effect was partially mitigated (area 5.22 ± 0.88 µm^2^, S/V = 2.18 ± 0.21), likely due to the additional energy substrate supporting cell homeostasis.

In contrast to NiO NPs, CuO NPs in combination with (−)-isopulegol caused the greatest increase in cell area (7.49 ± 3.58 µm^2^) with a minimum S/V ratio (1.98 ± 0.49). The increase in cell area (Table 1), combined with ultrastructural changes (plasmolysis, lysis, the corresponding results are presented below), indicates impaired cell wall barrier function under the action of CuO NPs. This is likely due to the generation of reactive oxygen species via a Fenton-like reaction initiated by Cu^2+^ ions released from the NPs [26]. Importantly, CuO NPs alone (without (−)-isopulegol) induced less pronounced changes (area 4.81 ± 1.52 µm^2^, S/V = 2.46 ± 0.37), suggesting a synergistic stress effect when CuO NPs and the monoterpenoid are combined.

Measurement of catalase activity in bacterial cells, which serves as one of the indicators of oxidative stress, revealed a significant increase in catalase activity on days 3–4 in the presence of CuO NPs, both alone and in combination with (−)-isopulegol (Figure 3). This confirms the cytotoxic effect of these NPs.

It is known that exposure to metal oxide NPs can lead to changes in the surface morphology of bacterial cells, including a decrease in cell wall roughness, which is attributed to the adsorption of NPs, the rearrangement of extracellular polymer components, or stress-induced compaction of surface structures [27]. In Ivshina et al. (2022), it was shown that the cell surface roughness in the presence of (−)-isopulegol was 4.37 ± 0.50 nm, and the zeta potential was −34.20 ± 0.37 mV, which was virtually indistinguishable from the biotic control [8]. This confirmed the absence of a damaging effect of the monoterpenoid on the cell wall. In the present study, the addition of metal oxide NPs led to considerably more pronounced changes (Table 2). NiO NPs reduced the roughness to 2.88 ± 0.53 nm and shifted the zeta potential to −15.95 ± 1.50 mV, i.e., towards less negative values. Under the combined action of NiO NPs and (−)-isopulegol, these changes were intensified: roughness dropped to 1.57 ± 0.60 nm, and the zeta potential was −18.87 ± 0.53 mV. The decrease in cell surface roughness in the presence of NiO NPs (Table 2) correlates with the appearance of NP aggregates on the cell wall (the corresponding results are presented below) and likely reflects the formation of a hybrid “cell wall–nanoparticle” layer. Such a layer may act as a trap for the hydrophobic substrate, increasing its local concentration in the vicinity of membrane-bound enzymes.

CuO NPs caused a less pronounced decrease in roughness (2.76 ± 0.89 nm) and a shift in zeta potential to −20.20 ± 0.25 mV. However, under combined action with (−)-isopulegol, these parameters stabilized (roughness 2.85 ± 0.74 nm, zeta potential −21.83 ± 0.69 mV), which may reflect the absence of CuO NPs adsorption on the cell surface.

It is known that an increase in zeta potential promotes enhanced cell aggregation [8,14]. In the presence of NiO and CuO NPs, phase-contrast microscopy (Figure 4) revealed the formation of large multicellular aggregates, which was especially pronounced in the variants with NPs and (−)-isopulegol.

Investigation of the respiratory activity of *R. rhodochrous* IEGM 1362 cells showed that its maximum occurred during the first three days of cultivation (Figure 5). The addition of NiO and CuO NPs led to a twofold decrease in respiratory activity, as well as an extended duration of the stationary phase. Moreover, the total amount of oxygen consumed in the presence of NPs was reduced approximately twofold.

Combined AFM-CLSM imaging (Figure 6 and Figure 7) offers a comprehensive elucidation of the physiological responses of *Rhodococcus* cells to metal oxide NPs, revealing a distinct divergence in their cytotoxic mechanisms. As evidenced by the 3D topographical reconstructions (Figure 7A,B,E,F), the adsorption of NiO NPs onto the cell wall elicits pronounced surface deformation and decreased roughness; however, concurrent Live/Dead staining demonstrates that a substantial fraction of the treated population retains membrane integrity, as indicated by sustained green fluorescence (Table S1). These observations are consistent with the moderate perturbations in zeta potential and confirm that NiO NPs primarily exert physical stress via surface accumulation while preserving the viability of the majority of cells. Conversely, the CuO NP-treated variants—particularly when challenged with (−)-isopulegol (Figure 7C,D)—exhibit a marked prevalence of red fluorescence, signifying extensive membrane permeabilization and cell death. Importantly, this cytotoxic outcome occurs in the absence of notable NP adsorption, as corroborated by AFM data, and strictly aligns with TEM findings of irreversible cell wall degradation and lysis (Figure 8C,D). Therefore, the combined data suggest that while NiO NPs induce a localized, surface-mediated morphological alteration with limited acute toxicity, CuO NPs trigger a non-adsorptive, lytic cascade culminating in the complete loss of cell viability, an effect that is substantially potentiated by (−)-isopulegol.

Transmission electron microscopy (TEM) of ultrathin sections was used to conduct a comparative analysis of morphological changes in *R. rhodochrous* IEGM 1362 cells. Cells from the biotic control, as well as cells cultured in the presence of (−)-isopulegol, exhibited normal ultrastructure (Figure 8D,E). The introduction of NiO NPs led to the appearance of a clumpy (coarse) cytoplasmic texture and plasmolysis (Figure 8A). In samples with NiO NPs and (−)-isopulegol, cells forming capsule-like surface protrusions were additionally observed (Figure 8B). Upon exposure to CuO NPs, population heterogeneity was revealed: cells with clumpy cytoplasm, cells undergoing plasmolysis, non-dividing (inhibited) forms with a capsule, as well as lysed cells were present (Figure 8C,D). The greatest degree of destruction was observed upon combined exposure to CuO NPs and (−)-isopulegol, which was manifested by a significant number of disrupted cells (Figure 8C).

Confirmation of NiO NP adsorption on the cell surface was also obtained using scanning electron microscopy with energy-dispersive X-ray spectroscopy (SEM-EDX). The nickel content on non-stainable cells was 10 times higher (Figure 9). We hypothesize that the adsorption of NiO NPs on the surface of *R. rhodochrous* cells may modulate redox-dependent enzyme systems, leading to enhanced hydroxylating activity and simultaneous suppression of the epoxidase pathway. At the same time, copper was detected in trace amounts, and its content did not differ significantly from the control values.

Thus, summarizing the presented data, it can be concluded that the rational use of NiO NPs may provide multifactorial optimization of the biotransformation process of (−)-isopulegol. In contrast to NiO NPs, CuO NPs exhibit pronounced cytotoxic effects at the concentrations used, rendering them unsuitable for practical application in this biotechnological process. The obtained results may open new prospects for targeted modulation of the catalytic activity of actinomycetes of the species *Rhodococcus rhodochrous* using NPs of variable-valence metal oxides.

## 3. Materials and Methods

### 3.1. Bacterial Strain

*R. rhodochrous* IEGM 1362 was isolated from the Paltinskoye peat deposit, Perm region, Russia, and deposited in the Regional Specialized Collection of Alkanotrophic Microorganisms (acronym IEGM, WFCC number 285). The strain not only transforms (−)-isopulegol but also utilizes *n*-hexadecane as a sole carbon source, degrades dehydroabietic acid, transforms the monoterpenoids (−)-*cis*-carveol, (−)-*trans*-carveol, and (−)-L-carvone, exhibits resistance to Cr^6+^ (40.0 mM), Mo^6+^, and Zn^2+^ (5.0 mM), and produces exopolysaccharides (http://www.iegmcol.ru/strains/rhodoc/rhodoch/r_rhod1362.html, accessed on 19 August 2026).

### 3.2. Chemicals

All commercially available chemicals and solvents were of reagent quality and utilized without additional treatment unless stated otherwise. (−)-isopulegol (purity ≥ 98%, MW 154.25 g/mol, C_10_H_18_O, (1*R*,2*S*,5*R*)-5-methyl-2-(prop-1-en-2-yl)cyclohexan-1-ol, CAS No. 89-79-2, ${\left[\alpha \right]}_{D}^{31}$ = −21 (c = 0.4, CHCl_3_)) was obtained from Sigma-Aldrich (St. Louis, MO, USA). NPs of NiO and CuO were purchased from M9 (Tolyatti, Russia). The sizes and AFM images of NPs are shown in Figure S1.

### 3.3. Biotransformation of (−)-Isopulegol

The biotransformation process was carried out in 250 mL Erlenmeyer flasks containing 100 mL of RS mineral salt medium (g/L: K_2_HPO_4_—2.0; KH_2_PO_4_—2.0; KNO_3_—1.0; (NH_4_)_2_SO_4_—2.0; NaCl—1.0; MgSO_4_—0.2; CaCl_2_—0.02, FeCl_3_ × 7H_2_O—0.001), under shaking conditions (160 rpm) at 28 °C. The cultivation medium was supplemented with yeast extract (0.1 g/L) and a trace element solution prepared according to Postgate (0.1% *v*/*v*). (−)-Isopulegol was added at a final concentration of 0.025% *v*/*v*. NiO and CuO NPs were added at concentrations of 50 mg/L and 5 mg/L, respectively, after first dispersing them in a phosphate buffer (pH 7.0) and performing sonication of the aggregates with ultrasound for 2 min, amplitude 7.5 µm (Soniprep 150, MSE, London, UK).

Abiotic controls were performed under identical conditions but without bacterial cells to exclude chemical oxidation catalyzed by NPs.

### 3.4. Phase-Contrast and Fluorescent Microscopy

Cell suspensions were examined using either an Axio Imager M2 optical microscope (Carl Zeiss Microscopy GmbH, Jena, Germany) fitted with an Axiocam 506 Color camera, or an Olympus FV1000 confocal laser scanning microscope (Olympus Corporation, Tokyo, Japan). To distinguish viable from non-viable cells, the suspensions were treated with the Live/Dead^®^ BacLight™ Bacterial Viability Kit (Invitrogen, Carlsbad, CA, USA), following the manufacturer’s instructions for sample preparation and imaging mode.

### 3.5. Atomic Force and Confocal Laser Scanning Microscopy (AFM-CLSM)

The morphology of bacterial cells was studied with a hybrid scanning platform that integrated an Olympus FV 1000 confocal laser scanning microscope (CLSM) (Olympus Corporation, Tokyo, Japan) and an Asylum MFP-3D atomic force microscope (AFM) (Asylum Research, Santa Barbara, CA, USA). AFM imaging was carried out in air in semi-contact mode using an AC240TS silicon cantilever (resonance frequency 50–90 kHz, contact stiffness 0.5–4.4 N/m, probe curvature radius 9 nm). The resulting images were processed with the FV10-ASW 3.1 software (Olympus Corporation, Tokyo, Japan). For combined AFM-CLSM visualization, CLSM images were transferred into the AFM software package (Igor Pro 6/22A, Wave Metrics, Portland, OR, USA).

### 3.6. Scanning Electron Microscopy (SEM), Transmission Electron Microscopy (TEM), and Energy Dispersive X-Ray Spectroscopy (EDX) with Elemental Mapping

For SEM, bacterial cells were treated with 1.5% (*w*/*v*) glutaraldehyde, rinsed, applied onto coverslips, and allowed to dry in air. The coverslips were subsequently attached to stubs and sputter-coated with Au using a JFC-1100 ion sputter (Jeol, Tokyo, Japan). Images were acquired with a JSM-IT200 electron microscope (Jeol, Tokyo, Japan).

For TEM, thin sections were cut on an 8800 Ultrotome III (LKB-Produkter, Stockholm, Sweden) and counterstained with lead citrate. The ultrathin sections were visualized with a JEM-1400 electron microscope (Jeol, Tokyo, Japan). TEM-EDX measurements with elemental mapping were carried out on the same JEM-1400 instrument (Jeol, Tokyo, Japan) coupled to an energy dispersive X-ray analysis system (EDXA, Inca Energy-350, Oxford Instruments, Abingdon, UK) and operated at an accelerating voltage of 80 keV (tilt angle, 15°). EDX spectra and elemental maps were recorded with AZtec software (Oxford Instruments, Abingdon, UK).

SEM, TEM, and EDX analysis were performed at the Core Facility Center “UNIQEM Collection” at the Research Center of Biotechnology, Russian Academy of Sciences.

### 3.7. Respiratory Activity

Oxygen consumption rates and cumulative oxygen uptake by *Rhodococcus* cells were determined with a 10-channel Columbus Micro-Oxymax respirometer (Columbus Instruments, Columbus, OH, USA). The assays were carried out in 300 mL flasks filled with 100 mL of RS medium and maintained under agitation (300 rpm, 28 ± 2 °C) using an Ikamag^®^ RO10 power magnetic stirrer (IKA-Werke, Staufen, Germany).

### 3.8. Analytical Methods

To recover the residual (−)-isopulegol and its derivatives, the post-culture medium was acidified with 10% HCl and then mixed with an equal volume of ethyl acetate. Once the emulsion had separated, the organic layer was collected. To maximize recovery, the remaining aqueous phase was extracted twice more with ethyl acetate. The pooled ethyl acetate extracts were evaporated on a Laborota 4000 rotary evaporator (Heidolph, Schwabach, Germany). The concentrate was subsequently washed sequentially with 1% aqueous NaHCO_3_ and distilled water until a neutral pH (~7.0) was reached, and then dried over anhydrous Na_2_SO_4_. The purified residue was used for the subsequent analytical procedures.

Qualitative assessment was carried out by thin-layer chromatography (TLC) using Alugram^®^ Xtra SIL G/UV254 plates (Macherey-Nagel, Düren, Germany). Separation was achieved with a mobile phase composed of *n*-hexane and ethyl acetate in equal proportions (1:1, *v*/*v*). Following development, the plates were allowed to dry in air and then visualized by exposure to iodine vapor.

The biotransformation products of (−)-isopulegol were characterized by GC–MS. For identification purposes, compounds **2** and **3** prepared as described in [8] and epoxide **4** synthesized according to [28] were employed as reference standards. Measurements were carried out on an Agilent 7890A gas chromatograph interfaced with an Agilent 5975C quadrupole mass spectrometer detector (Agilent Technologies, Santa Clara, CA, USA). Separation was performed on an HP-5MS quartz capillary column (30 m × 0.25 mm i.d.). He (1 atm) was used as the carrier gas. The column temperature was ramped from 50 to 280 °C. Mass spectra were recorded over an *m*/*z* range of 50 to 700 and then compared either with the spectra of authentic **2**, **3**, and **4** or with reference spectra from the NIST08 database for compound identification. The percentage contents of the substances were determined as relative values within the resulting mixture. GC–MS analysis was performed at N.N. Vorozhtsov Novosibirsk Institute of Organic Chemistry of the Siberian Branch of the Russian Academy of Sciences.

### 3.9. Zeta Potential Measurement

The zeta potential of bacterial cells was assessed by dynamic light scattering on a ZetaSizer Nano ZS instrument (Malvern Instruments, Malvern, UK) using Malvern ZetaSizer software, v. 2.2 (Malvern Instruments, Malvern, UK). Prior to measurement, cells were rinsed twice and resuspended in 0.1 M KNO_3_ (pH 7.0) to an OD_600_ of 2.0. The measurements were performed at 25 °C (pH 7.0) in a U-shaped cuvette equipped with gold-plated electrodes.

### 3.10. Minimum Inhibition Concentrations of NPs

The minimum inhibitory concentration (MIC) of CuO and NiO NPs against *R. rhodochrous* IEGM 1362 was established using the twofold serial dilution procedure [29] in 96-well polystyrene microtiter plates. NPs dispersed in sodium phosphate buffer (pH 7.0) were introduced into wells containing mineral salt medium RS (g/L: K_2_HPO_4_—2.0; KH_2_PO_4_—2.0; KNO_3_—1.0; (NH_4_)_2_SO_4_—2.0; NaCl—1.0; MgSO_4_—0.2; CaCl_2_—0.02; FeCl_3_·7H_2_O—0.001), yeast extract (0.1 g/L), and Postgate’s trace elements (0.1%, *v*/*v*) at a starting concentration of 50 g/L, after which serial twofold dilutions were prepared. A 10 µL aliquot of bacterial suspension (2 × 10^6^ cells/mL) was then added to each well. The plates were kept at the optimal temperature of 28 °C for 24 h. Cell viability was evaluated by staining with iodonitrotetrazolium chloride (Sigma-Aldrich, St. Louis, MO, USA). The appearance of insoluble formazan and the corresponding purple coloration served as an indicator of actively respiring cells in the wells. To account for non-specific dye adsorption, NPs were also tested without the addition of cell culture.

### 3.11. Statistical Analysis

Statistical analyses were performed using StatSoft Academic software (StatSoft LLC, Moscow, Russia). Student’s *t*-test was used for pairwise comparisons of experimental groups with the corresponding control groups. A *p*-value < 0.05 was considered statistically significant.

## 4. Conclusions

This study demonstrates that NiO NPs at a concentration of 50 mg/L effectively enhanced the biotransformation of (−)-isopulegol by *Rhodococcus rhodochrous* IEGM 1362, increasing the total yield of the desired diol and hydroxy acid from 36.1% to 48.3% and, importantly, simultaneously reducing the formation of the unwanted (*S*)-epoxide byproduct. The modulatory effect is attributed to selective adsorption of NiO NPs onto the cell surface (confirmed by AFM and SEM-EDX), which alters surface properties, induces moderate oxidative stress, and possibly modulates the activity of redox-dependent enzymes. In contrast, CuO NPs induce irreversible cell damage, making them unsuitable for this bioprocess. These findings provide feasibility for NP-assisted optimization of monoterpenoid bioconversion and open new avenues for improving both yield and selectivity in *Rhodococcus*-mediated transformations.

## Figures and Tables

**Figure 1:**
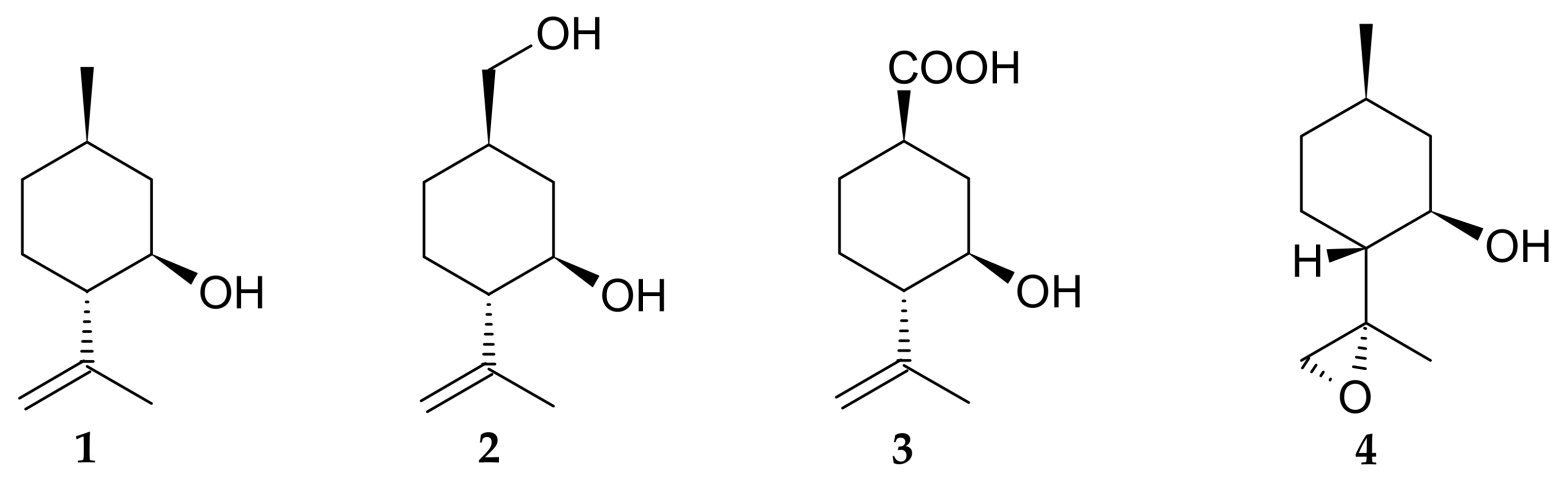
Chemical structures of (−)-isopulegol (**1**) and its biotransformation products (**2**–**4**).

**Figure 2:**
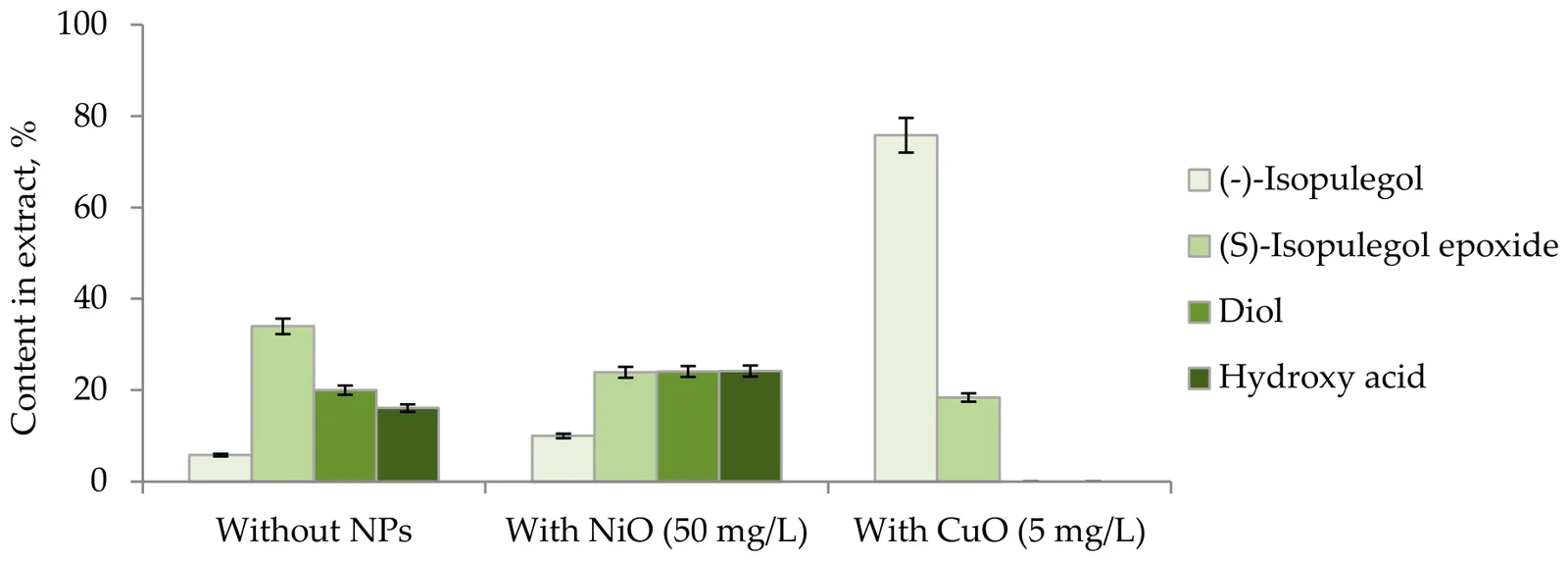
Biotransformation of (−)-isopulegol by *R. rhodochrous* IEGM 1362 cells in the presence of metal oxide NPs. Data are presented as mean ± SEM (n = 3).

**Figure 3:**
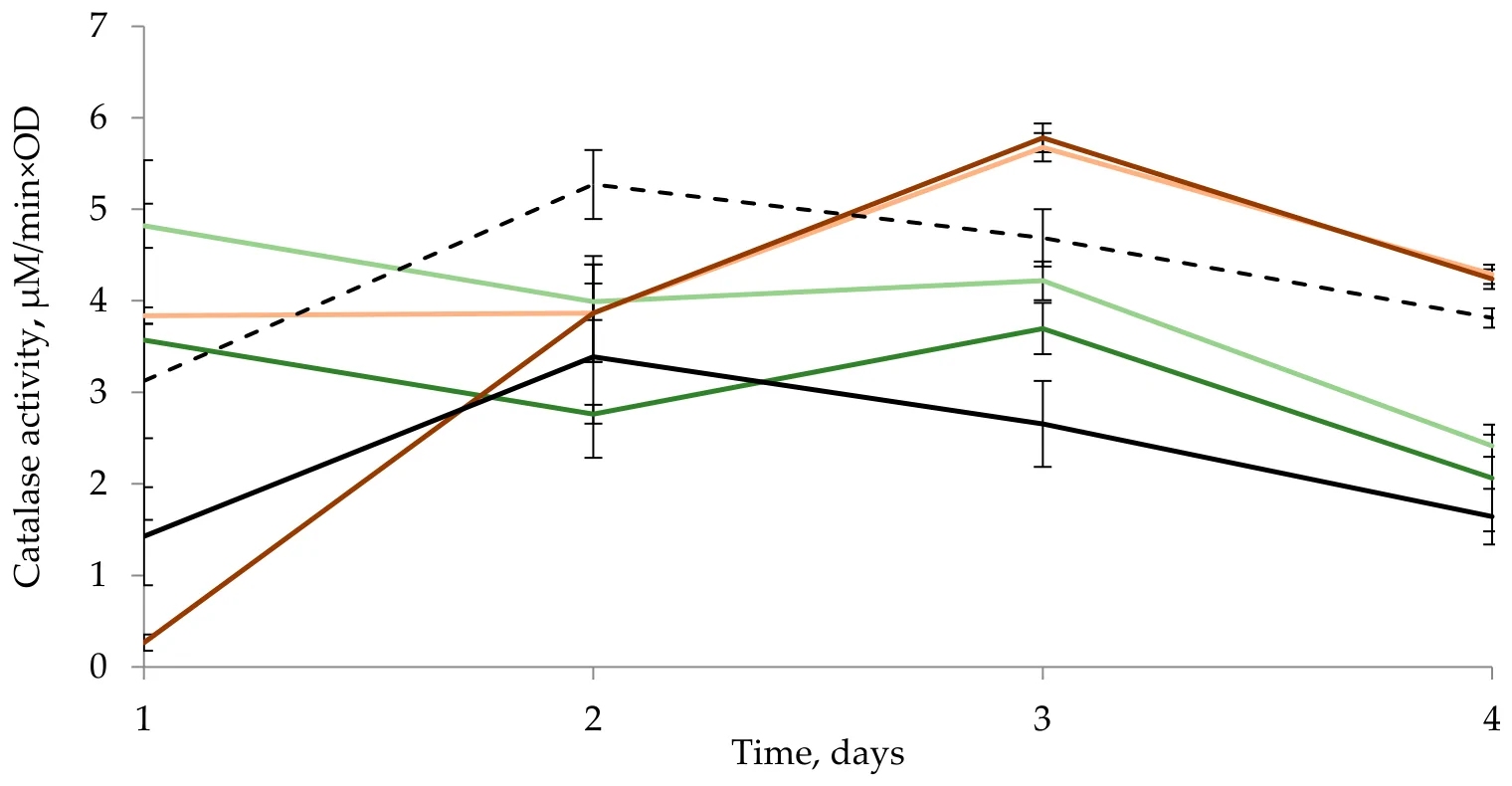
Dynamics of changes in *R. rhodochrous* IEGM 1362 cell catalase activity in the presence of (−)-isopulegol (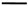), NiO NPs (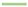), (−)-isopulegol and NiO NPs (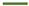), CuO NPs (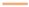), (−)-isopulegol and CuO NPs (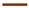). Biotic control (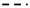). Data are presented as mean ± SEM (n = 5).

**Figure 4:**
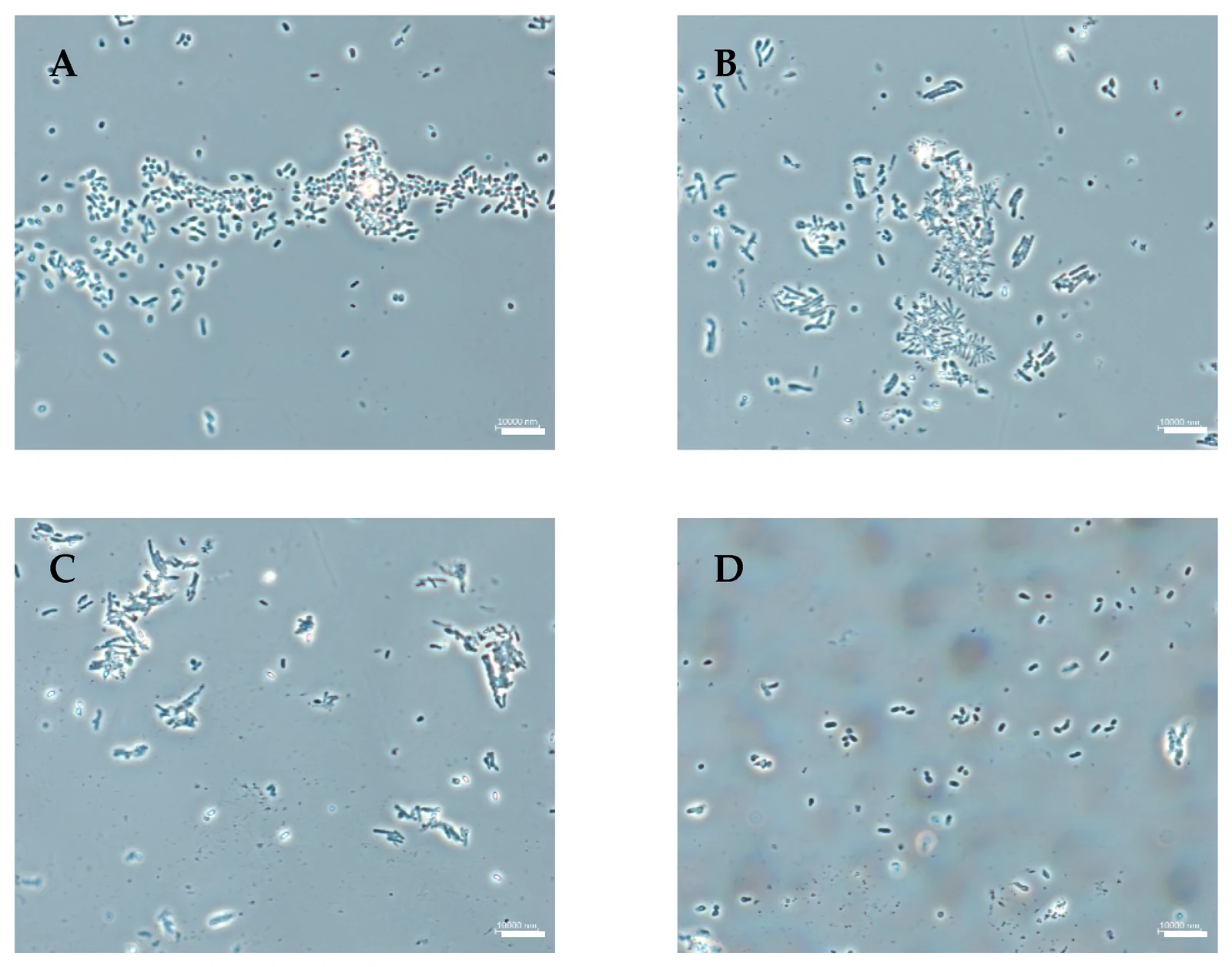
Phase-contrast microscopy (×1000 magnification) of *R. rhodochrous* IEGM 1362 cells in the presence of NiO and (−)-isopulegol (**A**), CuO and (−)-isopulegol (**B**), (−)-isopulegol (**C**). Biotic control (**D**). Scale bar is 10 µm.

**Figure 5:**
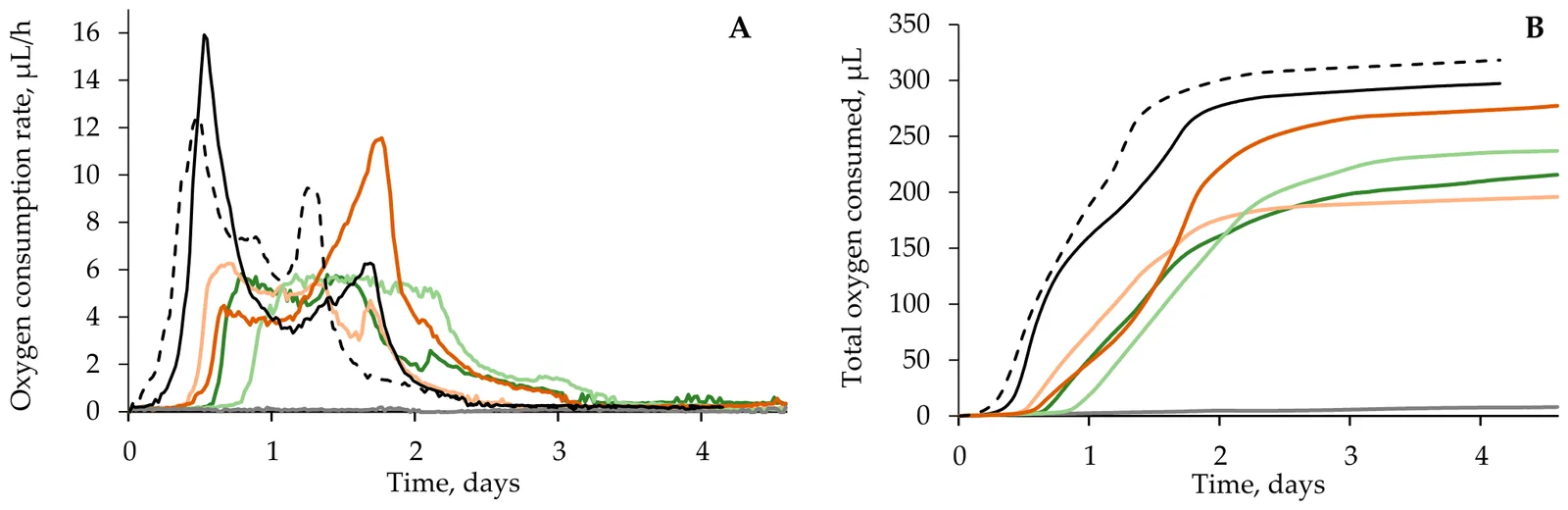
Dynamics of the oxygen consumption rate (**A**) and total oxygen consumed (**B**) by *R. rhodochrous* IEGM 1362 cells in the presence of (−)-isopulegol (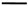), NiO NPs (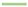), (−)-isopulegol and NiO NPs (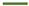), CuO NPs (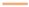), (−)-isopulegol and CuO NPs (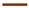). Biotic control (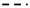), abiotic control (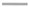).

**Figure 6:**
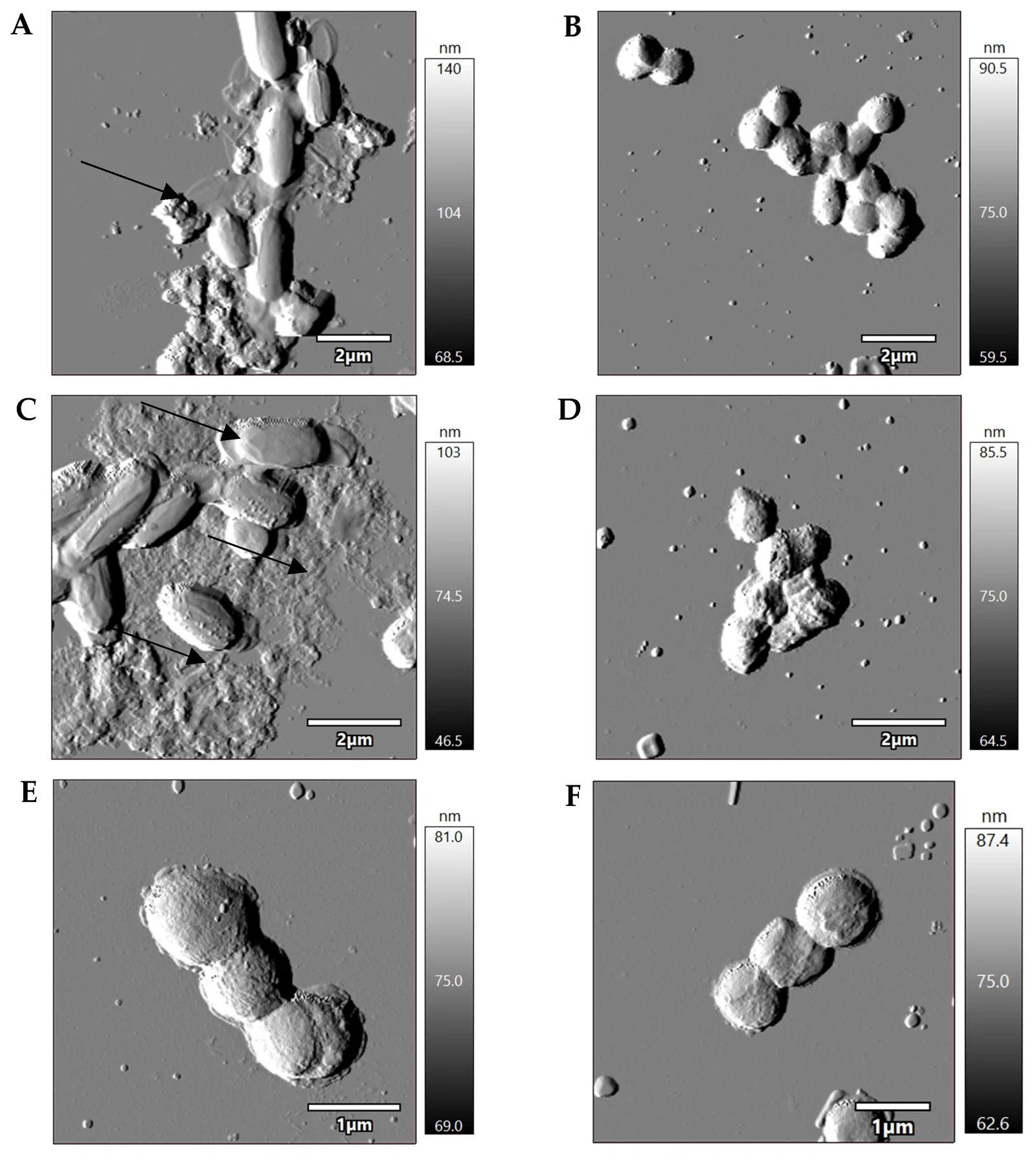
AFM images of *R. rhodochrous* IEGM 1362 cells in the presence of NiO NPs and (−)-isopulegol (**A**), CuO NPs and (−)-isopulegol (**B**), NiO NPs (**C**), CuO NPs (**D**), (−)-isopulegol (**E**). Biotic control (**F**). Arrows indicate accumulations of metal oxide NPs.

**Figure 7:**
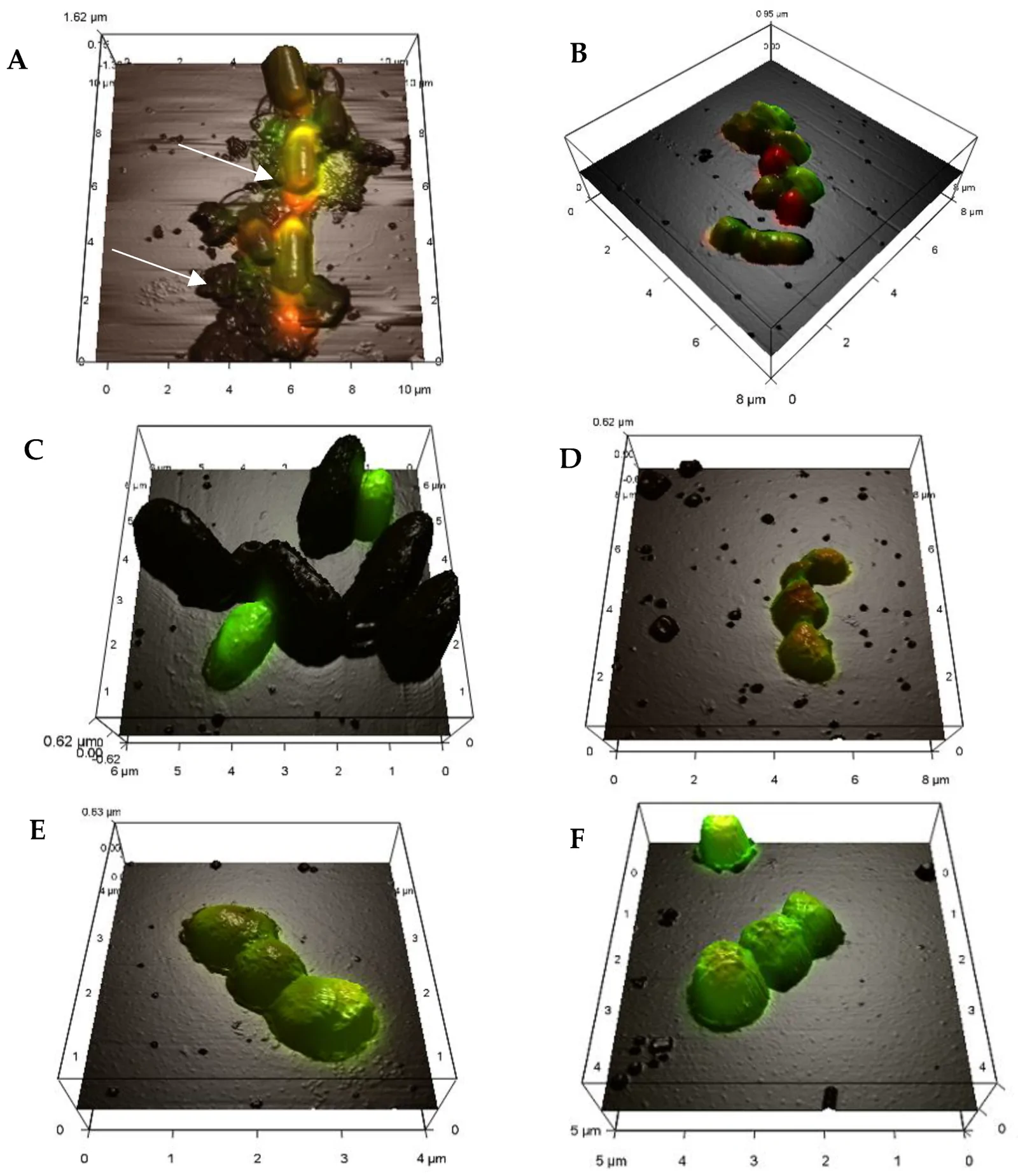
Combined AFM-CLSM images of *R. rhodochrous* IEGM 1362 cells in the presence of NiO NPs and (−)-isopulegol (**A**), CuO NPs and (−)-isopulegol (**B**), NiO NPs (**C**), CuO NPs (**D**), (−)-isopulegol (**E**). Biotic control (**F**). Dead cells are stained red; live cells are stained green. Arrows indicate accumulations of metal oxide NPs.

**Figure 8:**
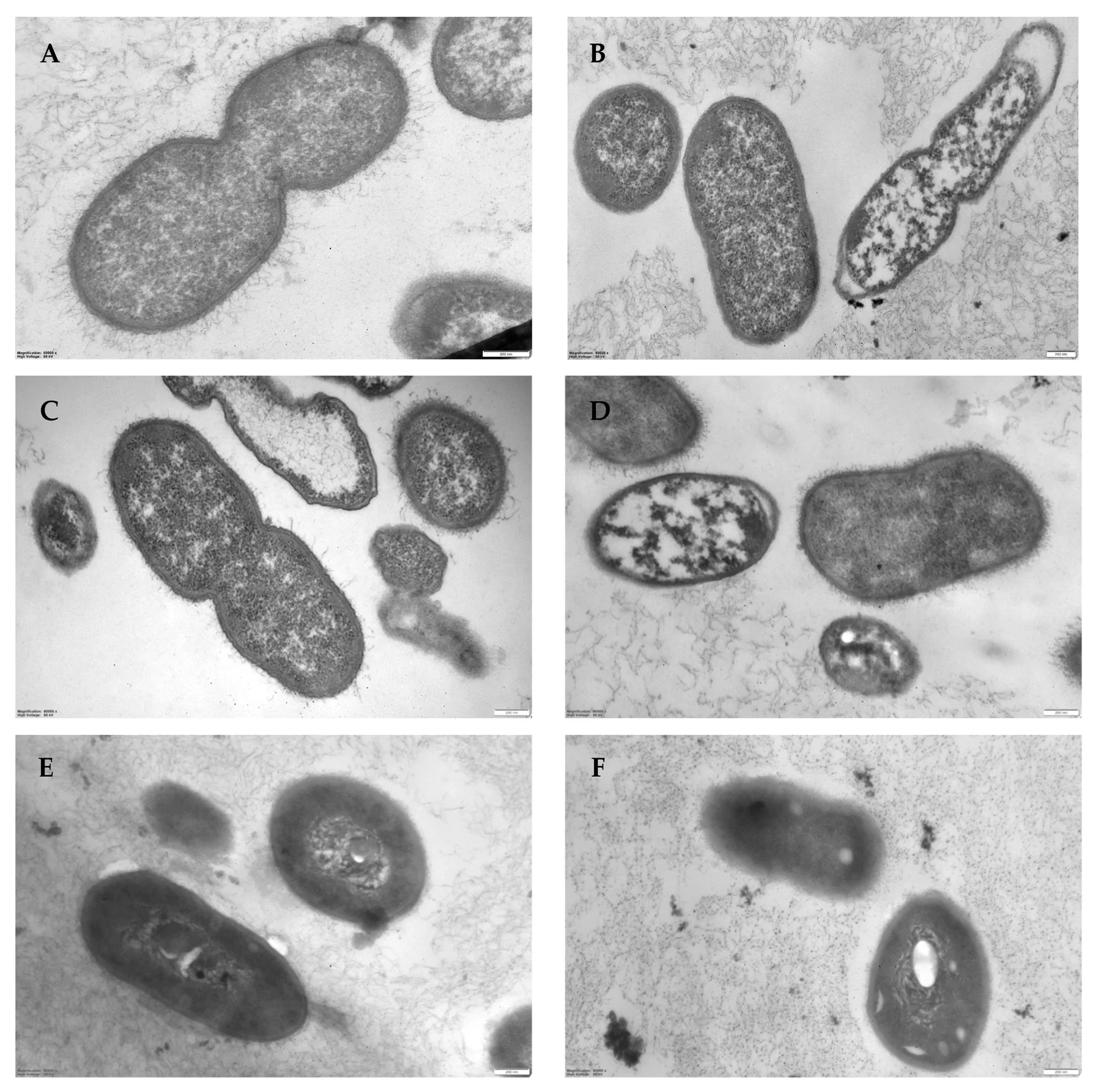
TEM of ultrathin sections of *R. rhodochrous* IEGM 1362 cells in the presence of NiO NPs and (−)-isopulegol (**A**), NiO NPs (**B**), CuO NPs and (−)-isopulegol (**C**), CuO NPs (**D**), (−)-isopulegol (**E**). Biotic control (**F**). Scale bar is 200 nm.

**Figure 9:**
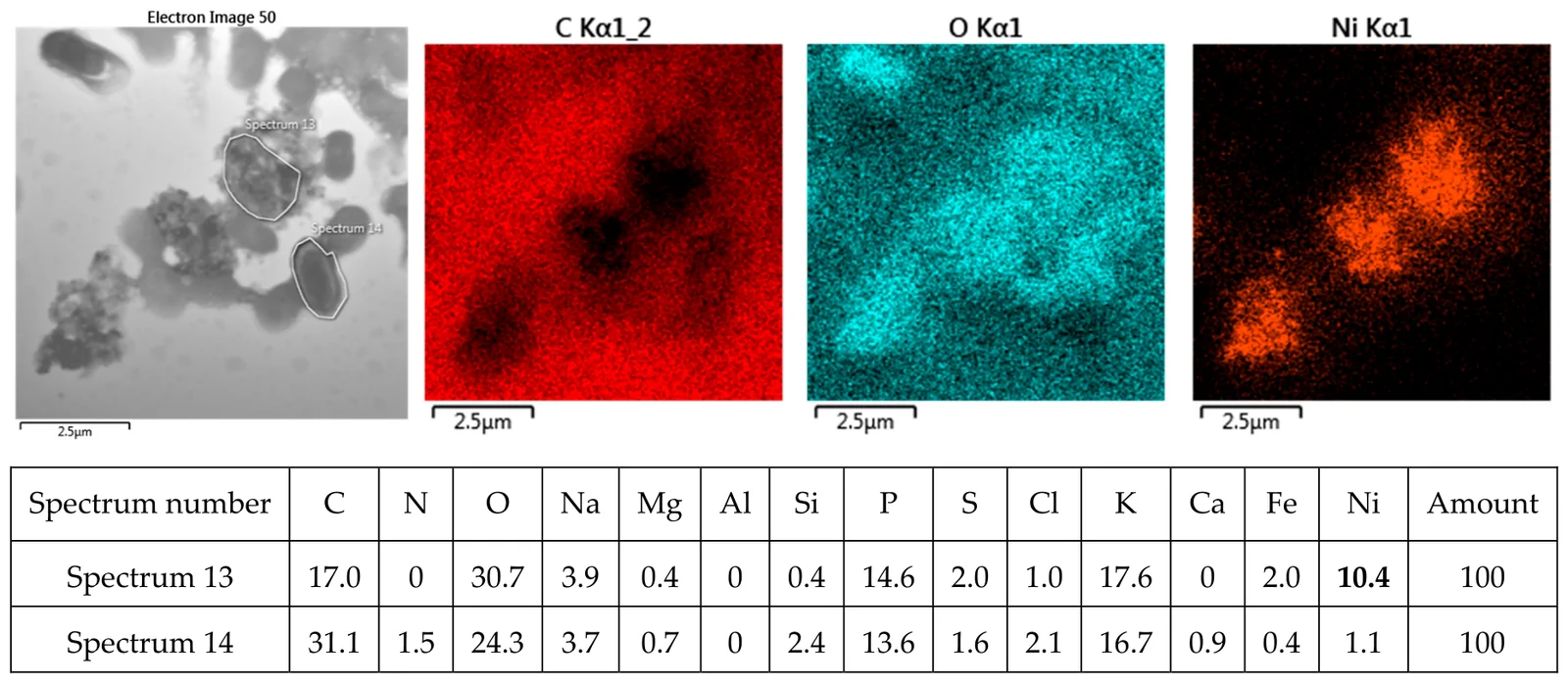
SEM-EDX analysis of *R. rhodochrous IEGM 1362* cells in the presence of NiO NPs and (−)-isopulegol.

**Table 1:** Effect of metal oxide NPs and (−)-isopulegol on the morphometric parameters of bacterial cells. Data are presented as mean ± SEM (n = 10).

Cultivation Conditions	Length, µm	Width, µm	Area (S), µm^2^	Volume (V), µm^3^	Relative Area (S/V), µm^−1^
In the presence of (−)-isopulegol and NiO	1.99 ± 0.43	0.88 ± 0.08	5.22 ± 0.88	2.44 ± 0.66	2.18 ± 0.21
In the presence of NiO	1.44 ± 0.23	0.72 ± 0.18	3.32 ± 1.24	1.22 ± 0.59	2.88 ± 0.45
In the presence of (−)-isopulegol and CuO	2.2 ± 0.58	1.05 ± 0.3	7.49 ± 3.58	4.35 ± 2.76	1.98 ± 0.49
In the presence of CuO	1.72 ± 0.48	0.89 ± 0.87	4.81 ± 1.52	2.06 ± 0.93	2.46 ± 0.37
In the presence of (−)-isopulegol	2.38 ± 0.55	0.92 ± 0.16	6.19 ± 1.92	3.31 ± 1.47	2.01 ± 0.37
Biotic control	1.51 ± 0.53	0.9 ± 0.17	5.56 ± 2.59	2.12 ± 1.53	3.01 ± 1.11

**Table 2:** Effect of metal oxide NPs and (−)-isopulegol on the cell surface properties of *R. rhodochrous* IEGM 1362. Data are presented as mean ± SEM (n = 10).

Cultivation Conditions	Roughness, nm	Zeta Potential, mV
In the presence of (−)-isopulegol and NiO	1.57 ± 0.6	−18.87 ± 0.53
In the presence of NiO	2.88 ± 0.53	−15.95 ± 1.5
In the presence of (−)-isopulegol and CuO	2.85 ± 0.74	−21.83 ± 0.69
In the presence of CuO	2.76 ± 0.89	−20.2 ± 0.25
In the presence of (−)-isopulegol	3.09 ± 0.52	−26.51 ± 0.05
Biotic control	4.37 ± 0.5	−34.20 ± 0.37

## Data Availability

The data presented in this study can be obtained on request from the corresponding author.

## Supplementary Materials

The following supporting information can be downloaded at: https://www.mdpi.com/article/10.3390/molecules31193445/s1, Table S1: The number of CFUs of *R. rhodochrous* IEGM 1362; Figure S1: AFM-images of NiO NPs (A) and CuO NPs (B). Sizes of NPs were measured in Igor Pro 6/22A software. Data are presented as mean ± SEM (n = 10).

## Abbreviations

The following abbreviations are used in this manuscript:

AFM	atomic force microscopy
CLSM	confocal laser scanning microscopy
EDX	energy-dispersive X-ray spectroscopy
GC-MS	gas chromatography-mass spectrometry
MIC	minimum inhibitory concentration
NPs	nanoparticles
SEM	scanning electron microscopy
TEM	transmission electron microscopy
TLC	thin-layer chromatography
