# Supplementary material for: Application of Metal Oxide Nanoparticles for the Bacterial Transformation of (−)-Isopulegol
Source: Molecules. 2026 Sep 27;31(19):3445. doi: 10.3390/molecules31193445 (PMC13665995; doi:10.3390/molecules31193445)
Supplement: Supplementary file 1 [file molecules-31-03445-s001.zip › molecules-4560854-supplementary.pdf]

The number of colony-forming units (CFUs) was determined by a micromethod. After 5 days of cultivation, bacterial suspensions were serially diluted tenfold, and 10 droplets of 5 μL each were plated per plate (one per sector) in 20 replicates using dilutions $1:10^3$, $1:10^4$, $1:10^5$, and $1:10^6$. CFU/mL was calculated as:

$$M = (\bar{x} \pm t_p \times S_{\bar{x}}) \times K \times \frac{1}{V}$$

where $\bar{x}$ is the mean number of colonies per droplet; $t_p$=2.1 is the Student's coefficient (95%, n=20); $S_x = {}^{\delta}/_{\sqrt{n}}$ is the standard deviation of the mean; $\delta = \sqrt{\sum P\,(x_i - \overline{x})^{2}/(n-1)}$ is the mean square error of a single measurement; $K$ is the dilution factor; and $V$ is the droplet volume.

**Table S1.** The number of CFUs of *R. rhodochrous* IEGM 1362.

| **Cultivation conditions** | **CFUs/mL** |
|---|---|
| In the presence of (–)-isopulegol and NiO | $(0.49\pm0.03)\times10^9$ |
| In the presence of NiO | $(0.69\pm0.05)\times10^9$ |
| In the presence of (–)-isopulegol and CuO | $(0.23\pm0.04)\times10^9$ |
| In the presence of CuO | $(0.32\pm0.06)\times10^9$ |
| In the presence of (–)-isopulegol | $(2.09\pm0.32)\times 10^9$ |
| Biotic control | $(4.87\pm1.66)\times10^9$ |

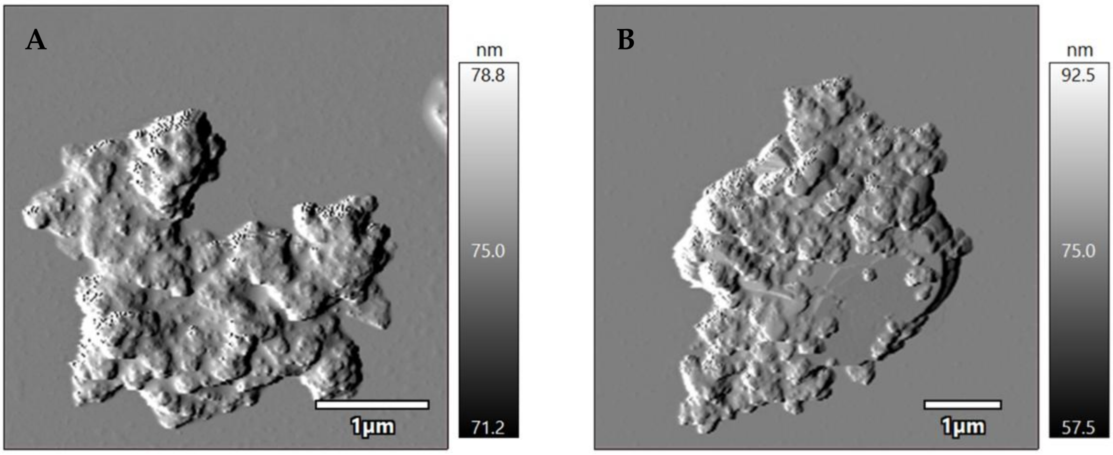


| | | | |
|---|---|---|---|
| Length, nm | 130.5±26.35 | Length, nm | 41±11.77 |
| Width, nm | 99.74±16.21 | Width, nm | 26.37±9.76 |

**Figure S1.** AFM-images of NiO NPs (**A**) and CuO NPs (**B**). Sizes of NPs were measured in Igor Pro 6/22A software. Data are presented as mean ± SEM (n = 10).
